# A Retrospective Cohort Study of Decompressive Techniques for Cubital Tunnel Syndrome: In Situ Decompression Versus Ulnar Nerve Transposition

**DOI:** 10.7759/cureus.74953

**Published:** 2024-12-02

**Authors:** Saran Malisorn

**Affiliations:** 1 Orthopaedics, Naresuan University, Phitsanulok, THA

**Keywords:** cubital neuropathy, cubital tunnel syndrome, elbow pain, paresthesia, ulnar nerve

## Abstract

Background: Cubital tunnel syndrome (CuTS) is the second most common nerve entrapment syndrome of the upper extremity after carpal tunnel syndrome. In situ decompression (ISD) and ulnar nerve transposition (UNT) are the major surgery methods in practice for the treatment of CuTS. However, controversies exist over the efficacy and safety of these methods.

Aim: The objective of the study was to compare the short- and long-term clinical outcomes in patients treated with ISD and UNT surgery.

Method: This was a retrospective study comprising 56 patients who underwent either ISD or UNT at Naresuan University Hospital between January 2013 and December 2022. Data on age, sex, hand involved, duration of the surgery, duration of the symptoms including the clinical outcomes such as elbow range of motion (ROM), quick disabilities of the arm, shoulder, and hand (QuickDASH), McGowan grade (MGG), Visual Analog Scale (VAS) for pain scores, motor and sensory conduction velocity (MCV1, MCV2, and SCV), and Tinel's sign were collected during the two-week, one-month, three-month, and six-month follow-ups post-surgery.

Results: Of the 56 patients in the study, 62.5% were female. The number of patients aged <60 years was higher in both groups. The mean age of patients in the ISD group was 49.07±6.13 years, while in the UNT group, it was 51.5±7.04 years. After one month, 53.57% and 32.14% of the patients recovered to MGG 1 in the ISD and UNT groups, respectively. The majority of the patients had MGG 1 six months after the surgery. The QuickDASH score and pain scores of the patients improved during all follow-ups, but no statistical significance was revealed when the two techniques were compared.

Conclusion: The study found that both the ISD and UNT were efficient and safe in treating CuTS. Further study with the inclusion of parameters such as revision surgery and complications would be vital.

## Introduction

Cubital tunnel syndrome (CuTS) or ulnar nerve compression is the second most common nerve entrapment syndrome of the upper extremity after carpal tunnel syndrome. Its prevalence is reported to be 1.8-5.9% in the United States, but not much data is available globally [1]. Any activities with excessive flexion and extension of the elbow, for example, boxing or punching, may cause CuTS [2,3]. Intermittent numbness and tingling in the ulnar ring and small fingers are the most common symptoms [4]. Depending on the severity of compression, CuTS causes weakness of the intrinsic muscles leading to loss of dexterity including a decrease in grip and pinch strength and pain of the medial elbow, forearm, or wrist [5]. The investigation of signs and symptoms and orthopedic and electrophysiological tests are crucial in the diagnosis of the condition.

The treatments of CuTS are based on the site of entrapment, but it occurs mostly in the inner posterior part of the elbow. Physiotherapy, bracing, and injection are the nonsurgical treatments [6]. However, a surgical release of the nerve at the site of compression is required in the severe stage. Surgeries such as in situ decompression (ISD) and medial epicondylectomy can provide symptomatic relief in the majority of patients [6,7]. Moreover, a surgical release may be done alone or with ulnar nerve transposition (UNT) through either subcutaneous, intramuscular, or submuscular anterior transposition at the elbow [8]. In advanced stages of ulnar nerve entrapment, UNT is regarded as the optimal intervention, with 75-90% positive outcomes. However, it is frequently associated with complications [6,9] and may eventually require correction by ISD. Unfortunately, clear information is not available regarding the best surgical method for CuTS. Some reports have mentioned that simple decompression is as effective as transposition and has a similar success rate and fewer complication rates [7,10]. The findings to support the best method for CuTS treatment are inevitable at the present time.

Therefore, the current study aimed to compare the long- and short-term clinical outcomes between ISD and UNT surgery which are the widely used treatments in patients with CuTS. Since there are no clear indications for one method over the other, the current findings will aid in suggesting a better surgical approach for the condition.

## Materials and methods

Study design

This was a retrospective study comprising 56 patients who underwent either ISD or UNT at Naresuan University Hospital between January 2013 and December 2022. The inclusion criteria were adults over 20 years of age who had CuTS assessed by electromyography. On the other hand, patients allergic to amoxicillin and xylocaine and with missing follow-up data were excluded. The study was approved by the Institutional Review Board of Naresuan University (approval number: 013/2023) and was carried out in compliance with international guidelines for human research protection such as the Declaration of Helsinki, Belmont Report, Council for International Organizations of Medical Sciences (CIOMS), and International Council for Harmonisation (ICH)-Good Clinical Practice (GCP).

Sample size

The sample size was calculated using a group-comparison (two-group) proportion formula based on a previous study [11]. After setting the type II error at 0.84 and the confidence level of 95%, the estimated sample size of the study was 56 (28 per group).

Procedure and data collection

The information was collected from the medical records and electronic database of the hospital. Previously, the patients were operated on by either of the surgery techniques under anesthetic conditions. By relieving pressure on the ulnar nerve and maintaining its natural anatomical position, the surgical technique known as ISD treats CuTS. The surgical technique known as UNT treats CuTS by relocating the ulnar nerve from its original position in the cubital tunnel to a new location in front of the elbow. The same surgeon carried out the two surgical procedures in both groups. The wound dressing was changed every day, and the patients were prescribed amoxicillin-clavulanate and analgesics. On the third day after surgery, an arm sling was advised for two days. Sutures were removed on the 12th to 14th day.

Data on age, sex, hand involved, duration of the surgery, duration of the symptoms including the clinical outcomes such as elbow range of motion (ROM), quick disabilities of the arm, shoulder, and hand (QuickDASH), McGowan grade (MGG), Visual Analog Scale (VAS) for pain scores, motor and sensory conduction velocity (MCV1, MCV2, and SCV), and Tinel's sign were collected during the two-week, one-month, three-month, and six-month follow-ups post-surgery.

Data analysis

SPSS Statistics for Windows, Version 17.0 (Released 2008; SPSS Inc., Chicago, Illinois, United States) was used for the analyses. Descriptive data were analyzed as frequency, percentage, mean, and standard deviation. Continuous variables in two groups were compared by Student's t-test, and categorical variables were analyzed using the chi-squared test. A p-value <0.05 was considered statistically significant.

## Results

Of the 56 patients in the study, the ISD group had a higher proportion of female patients (71.43%) compared to the UNT group (53.57%). The difference in gender among the patients who underwent ISD and UNT was statistically not significant (p=0.16). All patients in the ISD group were <60 years old, whereas 10.71% of patients in the UNT group were >60 years old. The mean age was slightly higher in the UNT group (51.5±7.04 years) compared to the ISD group (49.07±6.13 years). The patients <60 years old were higher in both groups without reaching a statistical significance (p=0.07). The majority of patients in both groups had right-hand involvement (ISD: 75%; UNT: 82.14%). The difference in hand involvement was not statistically significant (p=0.51). The operation time was significantly shorter for the ISD group (40.21±4.21 minutes) compared to the UNT group (66.28±5.61 minutes; p<0.01). Table 1 highlights that the demographic characteristics were comparable between the two groups, except for the operation time, which was significantly longer in the UNT group.

**Table 1:** Characteristics of the patients in the study Values are presented as N (%) or mean±SD. P-values were calculated using the chi-squared test for categorical variables and Student's t-test for continuous variables ISD: in situ decompression; UNT: ulnar nerve transposition; SD: standard deviation

Variables	ISD (n=28)	UNT (n=28)	P-value
Gender			
Male	8 (28.57%)	13 (46.43%)	0.16
Female	20 (71.43%)	15 (53.57%)	
Age (years)			
<60	28 (100%)	25 (89.29%)	0.07
>60	0 (0%)	3 (10.71%)	
Age (years)	49.07±6.13	51.5±7.04	
Hand side			
Left	7 (25%)	5 (17.86%)	0.51
Right	21 (75%)	23 (82.14%)	
Operation time (min)	40.21±4.21	66.28±5.61	<0.01

The mean duration of symptoms between ISD and UNT patient groups were 6.96±2.31 and 7.78±2.31 months, respectively (p=0.19). Likewise, the elbow ROM was similar between the two groups of patients (104.64±6.92 vs 103.92±6.85; p=0.69). According to the modified McGowan score, 53.57% of the patients in the ISD group were in grade 2A, whereas 50% in the UNT group were in 2B. The overall difference in terms of the grade was not significant (p=0.16). The motor conduction velocity across the elbow (MCV1) and forearm segments (MCV2) of the ulnar nerve and sensory conduction velocity (SCV) in the hand were similar between the two study groups (Table 2). Tinel's sign was positive for all the patients in either group. The majority of the patients who underwent ISD (89.29%) and UNT (78.57%) had a QuickDASH score of 4. The QuickDASH score, VAS, VAS face, and muscle power were significantly not different between the two surgery groups.

**Table 2:** Characteristics of the patients before the surgery For the McGowan grade, grade 1 indicates mild symptoms, grade 2A indicates moderate symptoms with intermittent weakness, grade 2B indicates significant sensory loss and moderate weakness, and grade 3 indicates severe symptoms with pronounced muscle weakness or atrophy. QuickDASH levels range from 0 (no disability) to 100 (most severe disability). VAS scores range from 0 to 10. Muscle power scores range from 0 to 5. P-values were calculated using the chi-squared test for categorical variables and Student's t-test for continuous variables ISD: in situ decompression; UNT: ulnar nerve transposition; elbow ROM: elbow range of motion; MG grade: McGowan grade; MCV1: motor conduction velocity across the elbow; MCV2: motor conduction velocity across the forearm segments of the ulnar nerve; SCV: sensory conduction velocity; QuickDASH: disabilities of the arm, shoulder, and hand; VAS: Visual Analog Scale

Characteristics	Before		P-value
	ISD (n=28)	UNT (n=28)	
Duration of symptom (months)	6.96±2.31	7.78±2.31	0.19
Elbow ROM (°)	104.64±6.92	103.92±6.85	0.69
MG grade			
2A	15 (53.57%)	8 (28.57%)	0.16
2B	9 (32.14%)	14 (50%)	
3	4 (14.29%)	6 (21.43%)	
MCV1 (m/s)	30.60±1.34	29.96±1.46	0.09
MCV2 (m/s)	40.82±1.72	40.38±2.09	0.40
SCV (m/s)	33.25±1.95	32.59±1.96	0.21
Tinel’s sign	28 (100%)	28 (100%)	1.00
QuickDASH			
4	25 (89.29%)	22 (78.57%)	0.27
5	3 (10.71%)	6 (21.43%)	
VAS	7.67±1.02	7.67±1.09	0.99
VAS face	3.35±0.62	3.35±0.78	0.99
Muscle power	3.85±0.35	3.78±0.41	0.49

During the two-week follow-up, the elbow ROM were similar between the patients in the two groups (107.14±5.99 vs 105.35±6.92; p=0.30) but increased after one month (117.5±6.45 vs 115.7±9.59; p=0.41). The McGowan grading of CuTS was not different between the patients who underwent ISD and UNT in both short-term follow-ups (p=0.67). However, an increase in MGG 2A and a decrease in MGG 3 were revealed among the patients two weeks after the surgery. After one month, 53.57% and 32.14% of the patients recovered to MGG 1 score in the ISD and UNT groups, respectively. None of the patients had MGG 3 at one month. Tinel's signs were similar after two weeks in both groups but reached a nearly significant level with lower frequency in patients treated with ISD compared to UNT (14.29% vs 35.71%; p=0.06). The QuickDASH score improved during the short-term follow-up after both surgeries, with no patients achieving a score of 5 by the second week and only one patient from the UNT group attaining a score of 4 at one month. The VAS score and VAS face score did not significantly change after two weeks and one month (Table 3).

**Table 3:** Clinical outcomes in the short-term follow-up For the McGowan grade, grade 1 indicates mild symptoms, grade 2A indicates moderate symptoms with intermittent weakness, grade 2B indicates significant sensory loss and moderate weakness, and grade 3 indicates severe symptoms with pronounced muscle weakness or atrophy. QuickDASH levels range from 0 (no disability) to 100 (most severe disability). VAS scores range from 0 to 10. Muscle power scores range from 0 to 5. P-values were calculated using the chi-squared test for categorical variables and Student's t-test for continuous variables ISD: in situ decompression; UNT: ulnar nerve transposition; elbow ROM: elbow range of motion; MG grade: McGowan grade; MCV1: motor conduction velocity across the elbow; MCV2: motor conduction velocity across the forearm segments of the ulnar nerve; SCV: sensory conduction velocity; QuickDASH: disabilities of the arm, shoulder, and hand; VAS: Visual Analog Scale

Variables	Two weeks		P-value	One month		P-value
	ISD (n=28)	UNT (n=28)		ISD (n=28)	UNT (n=28)	
Elbow ROM (°)	107.14±5.99	105.35±6.92	0.30	117.5±6.45	115.7±9.59	0.41
MG grade						
1			0.67	15 (53.57%)	9 (32.14%)	0.23
2A	16 (57.14%)	14 (50%)		10 (35.71%)	16 (57.14%)	
2B	10 (35.71%)	10 (35.71%)		3 (10.71%)	3 (10.71%)	
3	2 (7.14%)	4 (14.29%)		0	0	
MCV1 (m/s)	0	0		0	0	
MCV2 (m/s)	0	0		0	0	
SCV (m/s)	0	0		0	0	
Tinel’s sign	27 (96.43%)	26 (92.86%)	0.55	4 (14.29%)	10 (35.71%)	0.06
QuickDASH						
2			0.34	6 (21.43%)	4 (14.29%)	0.49
3	8 (28.57%)	5 (17.86%)		22 (78.57%)	23 (82.14%)	
4	20 (71.43%)	23 (82.14%)		0 (0%)	1 (3.57%)	
VAS	1.57±0.50	1.75±0.51	0.19	0.5±0.5	0.67±0.47	0.18
VAS face	0.64±0.48	0.64±0.48	1.0	0	0	NA
Muscle power	3.92±0.26	3.85±0.35	0.39	4.53±0.50	4.32±0.47	0.10

In the long-term follow-up at three and six months after ISD and UNT, the majority of the patients presented with MGG 1 (Table 4). Few presented with MGG 2B at three months but none in the sixth month. These findings were statistically not significant. Tinel's sign was positive in two patients with UNT surgery at three months but none at six months. The majority of the patients in both groups had QuickDASH scores of 2 at three months and 1 at six months. The scores were significantly not different. Likewise, the VAS score and muscle power were insignificant between the groups at three and six months of follow-up. 

**Table 4:** Clinical outcomes in the long-term follow-up For the McGowan grade, grade 1 indicates mild symptoms, grade 2A indicates moderate symptoms with intermittent weakness, grade 2B indicates significant sensory loss and moderate weakness, and grade 3 indicates severe symptoms with pronounced muscle weakness or atrophy. QuickDASH levels range from 0 (no disability) to 100 (most severe disability). VAS scores range from 0 to 10. Muscle power scores range from 0 to 5. P-values were calculated using the chi-squared test for categorical variables and Student's t-test for continuous variables ISD: in situ decompression; UNT: ulnar nerve transposition; elbow ROM: elbow range of motion; MG grade: McGowan grade; MCV1: motor conduction velocity across the elbow; MCV2: motor conduction velocity across the forearm segments of the ulnar nerve; SCV: sensory conduction velocity; QuickDASH: disabilities of the arm, shoulder, and hand; VAS: Visual Analog Scale

Variables	Three months		P-value	Six months		P-value
	ISD (n=28)	UNT (n=28)		ISD (n=28)	UNT (n=28)	
Elbow ROM (°)	127.85±6.86	127.14±9.75	0.75	141.78±9.04	138.92±10.65	0.28
MG grade						
1	20 (71.43%)	14 (50%)	0.25	26 (92.86%)	25 (89.29%)	0.63
2A	7 (25%)	12 (42.86%)		2 (7.14%)	3 (10.71%)	
2B	1 (3.57%)	2 (7.14%)		0	0	
3	0	0		0	0	
MCV1 (m/s)	0	0		0	0	
MCV2 (m/s)	0	0		0	0	
SCV (m/s)	0	0		0	0	
Tinel's sign	0	2 (7.14%)		0	0	
QuickDASH						
1	1 (3.57%)	0 (0%)	0.13	28 (100%)	25 (89.29%)	0.07
2	27 (96.43%)	25 (89.29%)		0	3 (10.71%)	
3	0 (0%)	3 (10.71%)		0	0	
4	0	0		0	0	
5	0	0		0	0	
VAS	0.03±0.18	0.14±0.35	0.16	0	0	
VAS face	0	0		0	0	
Muscle power	4.71±0.46	4.5±0.50	0.10	4.92±0.26	4.75±0.79	0.26

## Discussion

Various techniques are available for CuTS, which can be grouped into ISD and transposition procedures. The ideal surgical technique for the condition is highly debated. Each technique has been questioned over its efficiency and safety. Therefore, this study compared results between two widely used techniques for the treatment of CuTS: ISD and UNT. The results revealed that there was no superiority of one surgical procedure over the other.

The number of female patients in the study was insignificantly higher than that of male patients. Similar to our data, a previous study has reported more CuTS cases in women than in men below 50 years of age [12]. About 95% of patients in the study were less than 60 years old. It is to highlight that CuTS may develop in all age groups, but more profoundly in those with occupations requiring repetitive elbow movements and prolonged elbow flexion. The right hand was mostly affected in the study patients, which is possibly due to the high number of right-handed people than the left-handed in the global population. The surgery time in UNT was significantly higher than in ISD. This corroborates with a previously published study [13]. In general, ISD requires a smaller incision, thereby a shorter time.

It is to highlight that the duration of the symptoms in the study patients was similar; however, the majority (53.57%) in the ISD technique had MGG 2A, whereas 50% of the UNT surgery patients had MGG 2B. This could be possibly due to the surgeon's choice of a simple procedure with fewer complications for less severe CuTS [14]. Several factors affect a surgeon's choice of appropriate technique [15]. The similarity of patient characteristics between the two groups in terms of electrophysiological tests, VAS pain score, muscle power, and others before the surgery implies that the patients were matched and this certainly strengthens the outcomes of the study.

In the short-term follow-up, a higher percentage of the patients who underwent ISD recovered to MGG 1 compared to UNT at six months. This was true in terms of QuickDASH (score 2) as well. The almost significantly different result was found in fewer patients having positive Tinel's signs after ISD (p=0.06) than in the UNT surgery. At three months, a higher number of patients who had ISD recovered to MGG 1 than those who had UNT. At six months, about 90% of the patients were evaluated at MGG 1 in both groups. Likewise, the QuickDASH score was slightly better in ISD patients than UNT. Based on the short- and long-term clinical outcomes, it would be fair to mention that ISD was slightly better than UNT, which could be due to the fewer complications and the smaller incision in ISD as reported previously [16]. However, it is important to reiterate that the majority of the patients who underwent ISD in the study were initially at MGG 2A. Also, the sample size and retrospective nature of the study have to be considered while interpreting the results. The strengths of the study include a direct comparison of two established surgical techniques with validated outcome measures and comprehensive long-term follow-up. The limitations of the study include its retrospective design, its small sample size, and the lack of etiology-specific analysis for CuTS.

## Conclusions

The study finds that the two popular surgery methods, namely, ISD and UNT, produced excellent clinical outcomes in patients with CuTS. The ISD technique could be the better choice in the primary stage of ulnar nerve entrapment considering an early recovery in the study patients. Further study with the inclusion of parameters such as frequency of revision surgery and complications would be vital.
